# Intentions to “Reuse” Medication in the Future Modelled and Measured Using the Theory of Planned Behavior

**DOI:** 10.3390/pharmacy8040213

**Published:** 2020-11-12

**Authors:** Hamza Alhamad, Parastou Donyai

**Affiliations:** 1 Department of Pharmacy, University of Reading, Reading RG6 6AP, UK; 2 Department of Pharmacy, Zarqa University, Zarqa 132222, Jordan

**Keywords:** medicine reuse, theory of planned behavior, questionnaire, recycle, medicine waste, unused medicines, attitudes, intentions

## Abstract

Background: A range of pro-environmental behaviors are recognized, promoted, and investigated, but urgent action is also needed to tackle the direct and indirect environmental impact of medication waste. One solution is to reissue medicines, returned unused to pharmacies (i.e., reuse medicines). Yet, if medicines reuse is to be formally introduced in the UK, it is imperative also to understand people’s willingness to take part in such a scheme and importantly, the underpinning drivers. This study aimed to develop, validate, and evaluate a Theory of Planned Behavior model aimed at predicting medicines reuse behavioral intentions. Methods: The behavior of interest, medicines reuse, was defined according to its Target, Action, Context, and Time. Then themes from an existing qualitative study were used in order to draft, validate and pilot a Theory of Planned Behavior-based questionnaire before its completion by a representative sample (*n* = 1003) of participants from across the UK. Results: The majority expressed pro-medicines reuse intentions. The three direct measures accounted for 73.4% of the variance in relation to people’s intention to reuse medicines in the future, which was statistically significant at *p* < 0.001. People’s specific beliefs about medicines reuse and how they evaluate other people’s expectations of them had a substantial impact on their intentions to reuse medication in the future, mediated in an intricate way via attitudes, subjective norms and perceived behavioral control (PBC). Conclusions: This study shows how people could embrace medicines reuse via practical measures that illustrate the safety and quality assurance of reissued medicines, educational interventions that bolster beliefs about the pro-environmental benefits, and norm-based interventions encouraging doctors and pharmacists to endorse the practice. The findings add to the emerging work on medicines reuse and, significantly, provide a theoretical framework to guide policymakers and other organizations looking to decrease the impact of medication waste through medicines reuse schemes.

## 1. Introduction

Waste associated with prescribed medicines cost NHS England an estimated £300 million a year in 2009, £110 million of which related to medicines returned to community pharmacies for disposal [1]. A decade on, all medicines returned to community pharmacies in the UK continue to be branded as waste and earmarked for disposal [2]. Even if unused, unopened, and still in date, medicines thus returned are not allowed to re-enter the pharmaceutical supply chain, an often-cited reason being the possible loss of potency from unknown storage conditions outside a pharmacy [3] or the threat of tampering and falsification. However, some countries including the United States (US) [4] and Greece (GIVMED) [5] do operate “medication reuse” schemes, mainly for benevolent reasons—the idea being, briefly, that suitable medicines can re-enter the supply chain and be reissued to others in need. Furthermore, researchers have found that around 20–25% of medicines returned for disposal to community pharmacies in the UK and the Netherlands are potentially eligible for reuse [6,7], 90% of donations to a pilot recycling scheme in Singapore were reusable [8] and 10 million unused prescription medication discarded by long-term care facilities such as nursing homes in the US could be reused [9]. These studies form part of a global drive, including in the UK [10], to examine the evidence for medicines reuse as a plausible arrangement for tackling medication waste. Although an internationally agreed definition of medicines reuse remains to be developed, further description is outlined in a previous study [11]. 

Medicines reuse concurs with ideas on sustainable pharmacy [12] because, in addition to the economic impact of medication waste, accruing unused medicines can also produce deleterious environmental effects. For example, according to a systematic review [13], globally people are more likely to dispose of their unwanted residential medication in the household bin than use any other method, with disposal down the drain also taking place especially with liquid formulations and, in the US, with dangerous substances. These practices can contribute to water and ground contamination [14]. In fact, disposal via the drain is considered to be the most harmful route as it leads to direct input into the aquatic system via effluent released from wastewater treatment works [15] where pharmaceutical traces have been shown to withstand standard treatment methods [16]. The environmental impact of disposing of medicines into garbage also poses a risk especially where landfilling (versus, say, incineration) is a key waste management route (e.g., 42% of waste ends up in landfill in Ireland, and 35% in the UK) or where landfills act as dumping areas rather than being engineered sites (e.g., in Malaysia, Thailand, India, Bangladesh) [13]. Prescribed medication waste can also impact negatively on the environment through the “carbon footprint” [17]. Thus, logically reusing prescribed medicines could reduce the economic and environmental cost of medication waste provided a workable scheme exists, and people can be motivated to use it. 

In the UK the logistics of a credible scheme for reusing medication have been largely academic [18]. For patients, most NHS medicines are supplied free of charge or at a fixed cost (£9 per product in 2020) [19], weakening the humanitarian impetus. Regardless, the professional and regulatory bodies express no appetite to explore opportunities or a change in the law to enable medicines reuse, citing concerns over medicinal quality as the main rationale [10]. Yet, the UK government has relaxed its position on medicines reuse sporadically in the past, meaning the concept is not altogether implausible. In 2008 the Department of Health proposed the reuse of patient-returned and date-expired medicines when it anticipated supply-chain problems amid a flu pandemic [20]. More recently, in 2018, a drug company was allowed to extend the expiry date of adrenaline auto-injectors by four months, including for products already in circulation, when the supply chain was disrupted [21]. With medication shortages now a recognized problem in the UK [22], alongside the general urgency to halt the further environmental decline, a formal scheme for reusing medication becomes increasingly plausible. Yet, if medicines reuse is to be formally introduced, it is imperative to understand both pharmacist and people’s views and willingness about the medicine reuse idea. A UK study reported that pharmacists of one Health Board in South-East Wales are willing to redistribute returned unused medicines if certain criteria were met such as medicines being solid dosage forms only, with tamper evident seals in place [23]. This study focused on understanding people’s willingness to take part in such a scheme by accepting reissued medicines, and importantly, the underpinning drivers. There is evidence to suggest that environmental knowledge is positively linked with pro-environmental behavior regarding medicines disposal practices [24,25]. In addition, although a qualitative study in the UK suggests people could accept medicines in their original packaging if these are considered to be safe for reuse by the pharmacist [11], large-scale beliefs and intentions to personally take part in a medication reuse scheme in the future remain unexplored. 

This study aimed to address this gap by measuring people’s views on personally taking part in medicines reuse (by accepting reissued medicines) with the Theory of Planned Behavior (TPB) [26] as the underpinning framework. The TPB has been shown to be relevant to studying environmental problems and pro-environmental behaviors [27,28], including managing household waste and recycling [29,30]. According to the TPB, people’s intentions to personally take part in any particular behavior (e.g., smoking, eating healthy food, or, in this paper, taking part in medicines reuse) is the most proximal determinant of that behavior. In this model, behavioral intention is predicted by the psychological constructs “attitude” toward the behavior (which is a person’s overall evaluation of the behavior), “subjective norm” (which is a person’s own estimate of social pressure to perform the behavior or not), and “perceived behavioral control” (PBC) (the extent to which a person feels able to enact the behavior). Although each predictor of intention could be measured directly (by asking respondents, for example, about their overall attitude toward the behavior in question), the TPB approach to measurement also encompasses indirect questions. The indirect questions relating to *attitude*, measure specific beliefs about the likely consequences of the behavior (behavioral beliefs) and corresponding outcome evaluation for each behavioral belief. The indirect questions relating to the *subjective norm*, measure beliefs about how other important people would like them to behave (normative beliefs/social pressure) and their motivation to comply with each of these reference groups or individuals. The indirect questions relating to *PBC*, measure beliefs about how much a person has control over the behavior (control beliefs) and the power of the factors that facilitate or inhibit the behavior. The model has been shown to be robust and effective [31]. 

Taking account of these constructs, a Medicines Reuse Questionnaire (MRQ) was developed, validated and then used to survey a representative sample of just over 1000 people in the UK. We theorized:

**Hypothesis 1** **(H1).** There will be a positive relationship between medicines reuse attitudes and intention to reuse medication in the future.

**Hypothesis 2** **(H2).** There will be a positive relationship between medicines reuse subjective norms and intention to reuse medication in the future.

**Hypothesis 3** **(H3).** There will be a positive relationship between medicines reuse PBC and intention to reuse medication in the future.

**Hypothesis 4** **(H4).** There will be a positive relationship between medicines reuse behavioral beliefs and medicines reuse attitudes.

**Hypothesis 5** **(H5).** There will be a positive relationship between medicines reuse normative beliefs, and medicines reuse subjective norms.

**Hypothesis 6** **(H6).** There will be a positive relationship between medicines reuse control beliefs and medicines reuse PBC.

The rest of this paper is organized as follows. The methods for the development and validation of the MRQ are discussed as well as for the conduct of the survey. Then the descriptive and statistical analyses of the data are discussed followed by a wider discussion which includes the limitation of this work. The article concludes with a summary of the findings and final statements.

## 2. Materials and Methods 

### 2.1. Compliance with Ethical Standards

This study was approved by the University of Reading’s Research Ethics Committee through the School Exemptions process (reference number 30/15) with an amendment approved 2/2017.

### 2.2. Questionnaire Development

The development of a TPB questionnaire requires a methodical approach that unearths both the direct and indirect measures of intention. Accordingly, the MRQ was developed in eight stages consistent with the procedure set out by Francis et al. (2004), as briefly described below and shown in (Figure S1). 

First, the behavior of interest, taking part in medicines reuse, was defined in terms of its Target, Action, Context, and Time (TACT) [32]. Here the target was prescription medication previously given out to another individual but then returned to a pharmacy. The action was accepting medication for one’s own personal use. The context was a situation where the pharmacist has verified that the medication has been kept by the other individual for less than three months, has more than six months of shelf-life remaining, has not been tampered with, has been kept under normal storage conditions, and has been kept in an original sealed blister pack (i.e., medication strip). In addition, the time was in the future when collecting own medication from a pharmacy for the management of a long-term illness. See (Box S2) for the presentation of this information to the participants at the start of the MRQ. 

Second, to construct the indirect questions, the themes obtained from an elicitation study [11] were mapped against the constructs in the TPB model [32] in order to identify the behavioral beliefs, normative beliefs, and control beliefs. As medicines reuse does not currently take place in the UK, the normative beliefs encompassed injunctive norms only. Third, a draft questionnaire was produced to include items covering at all these constructs (see Figure S3). Likert scales for the indirect measures were unipolar and graded from 1 to 7 for the behavioral belief items, motivation to comply items and control belief items. The Likert scales were recoded into bipolar scales from -3 to +3 for the outcome evaluation items, normative belief items, and power of control factors, as per published recommendations [32]. The recoding allowed a composite score to be obtained for each of the indirect measures by multiplying scores on the relevant unipolar scale by those on the respective bipolar scale so that positive scores reflect favorable attitudes, more social pressure to perform the behavior (i.e., reuse medication), and control factors that make medicines reuse more likely, and vice versa with negative scores (see Tables S4–S6). 

In addition, 12 items in total were initially developed for the “direct” measures of attitude, subjective norm, and perceived behavioral control. Where appropriate, a mix of positive and negative endpoints was used to intermittently prompt respondents to further contemplate their answers. The endpoints of the direct measures of attitude were constructed using bipolar adjectives on a 7-point Likert scale with both instrumental (whether reusing medication achieves something, e.g., worthless-worthwhile) and experiential items (how it would feel to reuse medication, e.g., satisfying-dissatisfying, good-bad). The endpoints of the direct measures of subjective norms prompted respondents either to complete an otherwise incomplete sentence or to agree or disagree with a complete sentence. The endpoints of the direct measures of PBC were developed to assess the person’s “self-efficacy”, using both an incomplete sentence and a complete sentence prompting respondents to either agree or disagree, and to assess beliefs about the “controllability” of the behavior using complete sentences prompting respondents to either agree or disagree. The composite scores for each of the direct measures were obtained by calculating the mean of the item scores (see Tables S7–S9). Three items related directly to the intention construct were developed using a generalized intention method with positive (strongly disagree/strongly agree) endpoints. The composite score for the three intention items was obtained by calculating the mean score (see Table S10). Finally, seven items were developed to measure respondents’ demographic/background characteristics (i.e., age, gender, religion, ethnicity, the level of education, whether currently using medication and having any long-term conditions) using a multiple-choice response format. This generated 50 items initially. 

Fourth, the content validity (CV) (i.e., how well the items cover and assess the constructs of interest) of the first set of 50 questions was determined by interviewing a panel of 11 service users who had taken part in a previous elicitation study. This was carried out using the process of cognitive interviewing, which involved asking each participant four questions about each item on the questionnaire [33]. As a result, three of the items on the questionnaire (Q11, Q12, Q37) were reworded (see Table S11). This produced the first version (V1) of the MRQ (see Table S12). Fifth, MRQ (V1) was transferred to the Bristol Online Survey (as then known) platform and pilot tested with 46 participants recruited by emailing staff and students within the School of Pharmacy. Responses were subjected to construct validity analysis using confirmatory factor analysis (CFA) and the Statistical Package for Social Sciences (SPSS) software version 23. Data screening for multicollinearity and singularity was performed. To explain, first, principal component analysis (PCA) with orthogonal rotation (varimax), Bartlett’s test, and the Kaiser–Meyer–Olkin (KMO) measure were completed. The R matrix (the correlation matrix), an SPSS output produced using the coefficient and significant levels options, showed that multicollinearity was not a problem. Sampling adequacy was verified by an overall KMO of 0.735 (with all KMO values for the individual items being >0.59). Finally, the value of Bartlett’s test of sphericity χ^2^ (253) was = 1287.947, *p* < 0.001, indicating that correlations between the items were sufficiently large for completion of the analysis. As a result, CFA was performed with a sample of 46 responses using Analysis of a Moment Structures (Amos) SPSS software (v. 23). For the factor loadings for each item of the indirect and direct measures, see Table S12. Items with low factor loading were later deleted (Q38 and Q43 in MRQ V1) (a question pair relating to PBC) or rephrased (Q5, Q14, Q15, Q26, Q27, Q30, and Q31), as described below. 

In addition, two methods were used to measure the reliability of the MRQ items. Cronbach’s alpha (α) coefficient was used as a measure of internal consistency of the “direct” measures of TPB using the responses obtained from the 46 participants. The Cronbach’s α coefficient of the different constructs (relating to the direct measures) of the TPB are shown in Table S13. These were found to be excellent apart from the Cronbach’s α coefficient value of the PBC construct, which was below 0.5, but improved first by deleting Q15 (the Cronbach’s α value increased from 0.303 into 0.562), then further improved by also deleting Q14 (the Cronbach’s α value further increased from 0.562 into 0.830). These two questions related to PBC (“controllability”) and as noted above, were already flagged for rewording due to low factor loadings as part of CFA. Pearson correlation was used as a measure of the test-retest reliability of the “indirect” measures of TPB. Therefore, as a sixth step, 24 of the participants recompleted the same survey after two weeks, and this allowed the test-retest reliability of the indirect measures of MRQ (V1) to be determined (using Pearson’s coefficient) (see Table S12). Of the 28 items that were the indirect measures, 22 had correlations that met the threshold for reliability (>0.5). The Pearson correlation of 6 items (Q26, Q30, Q27, Q31, Q38, Q43) was <0.5. This provided further the rationale for removing two items (Q38, Q43) (a question pair relating to PBC) and rephrasing four items (Q26, Q27, Q30, Q31) as described below.

Seventh, MRQ (V1) was reworded by working with ten service users, recruited through the original elicitation study to produce the second version of the MRQ (V2). Two items (Q38, Q43) which related to the idea that a reward system may encourage people to reuse medicines in the future, had low factor loading (0.026) for the composite behavioral belief, and low Pearson’s correlation coefficients (<0.5). The service users expressed that a reward system would not affect their decision to reuse medicines. These two items were therefore removed. Four items for the composite normative beliefs (Q26, Q30 and Q27, Q31) with low factor loadings (0.358 and 0.356, respectively) and low Pearson’s correlation coefficients (<0.5) were reworded. These items related to normative beliefs (injunctive norms) and motivation to comply with the expectations of environmentalists and the pharmaceutical industry, respectively. On speaking with the service users about this, they expressed that their own decisions about medicines reuse would be affected by social pressure from pharmacists and doctors and not the groups listed above—Q26, Q30 and Q27, Q31 were modified accordingly. Finally, one item relating to the direct measures of subjective norms (Q5) and two items relating to the direct measures of PBC (Q14 and Q15) which all had low factor loadings (0.262, 0.258 and 0.019, respectively) were reworded with the help of the ten service users (see Table S12). 

Eight, MRQ (V2) was administered to a further set of 46 participants recruited by emailing staff and students from the wider university community. Responses for this second pilot were subjected again to validity (CFA) and reliability (internal consistency of the direct measures using Cronbach’s α) tests. The result of the second pilot showed good factor loading for all the items except for two items from the PBC construct (Q14, Q15) which measured the controllability of medicines reuse behavior (factor loading −0.068 and −0.052, respectively) (see Table S12). Also, the Cronbach’s α coefficient values of the direct measures of the TPB constructs were consistent and better compared to the previous pilot testing (see Table S14), again except for the PBC construct which had a Cronbach’s α coefficient value of only 0.425. As Q14 and Q15 had been reworded previously, they were deleted at this stage, meaning that the PBC items that remained measured only self-efficacy in relation to medicines reuse behavior. 

The above steps resulted in the refinement of MRQ (V3) with 46 items as a stable and accurate questionnaire ready for dissemination to a larger sample of the population. 

### 2.3. Questionnaire Distribution

The final version of the questionnaire, MRQ (V3), was transferred to the Qualtrics online platform and disseminated to a panel of participants via a market research company (Research Now^®^, Plano, TX, USA). The aim was to recruit a sample of over 1000 participants with a long-term health condition, from different regions in the UK, and across all age groups and genders. Thus, as well as using a sampling technique that targeted those with a long-term condition, three additional questions were added to MRQ (V3), namely: “We are interested in the views of people with a long-term health condition only, do you currently have a long-term health condition?”, “Which of the following (or another) long-term health condition(s) do you have?”, and “In which region of the UK do you currently live?”. The question about participants’ religion was removed as it was considered a sensitive question in hindsight and its inclusion could not be justified. 

The intended sample size was divided into participants with long-term conditions who were using medicines (*n* = 800), or not currently using medicines but had used medication for their condition in the past (*n* = 100) or had never taken any medicines for their long-term condition (*n* = 100). The recruitment was, therefore mainly targeting people with a long-term condition who were using medicines, as they might reasonably be expected to consider reusing medication in the future if the practice became a reality. However, it was important not to miss the viewpoints of those not presently using medicines (10% of the sample size), or those who have never taken any medicines for their long-term condition (10% of the sample size), as they too might require medicines in the future and thus have a view on medicines reuse.

A soft launch (10% of the total sample, *n* = 100) of MRQ (V3) was undertaken to review and quality-check the data before the full launch. Data obtained during the soft launch were included in the main study analysis. During the two-week data collection period, the representativeness of the sample was monitored for geographical spread, age groups, and gender balance, but no adjustment to the recruitment strategy was found to be necessary. Descriptive analysis was completed with the anonymized dataset using SPSS (V23).

### 2.4. Questionnaire Sample

**Figure 1:**
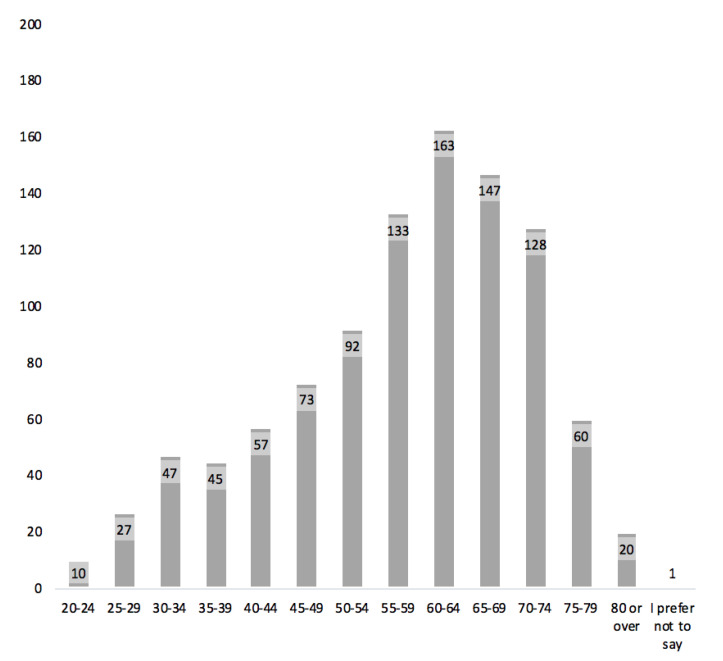
The number of participants in each age group.

A total of 1,181 people was invited to complete the MRQ (V3), with 178 potential respondents excluded because they reported not to have a long-term condition, resulting in 1003 useable responses. A summary of the background factors including gender, age, ethnicity, educational level, participants’ geographical areas in the UK, and if participants were taking medicines for their long-term conditions is all shown in Table S15, Supplementary material. There was an almost equal number of female (*n* = 509) to male (*n* = 494) participants. Figure 1 shows the age distribution of the participants. 

In addition, there was an excellent spread of responses from across the UK (see Table S15). The majority of the participants were taking medicines for their long-term condition (86.4%) (*n* = 867), 10% (*n* = 100) were not taking medicines but did take medicines in the past, and 3.6% (*n* = 36) were not taking medicines and had never taken any medicines for their long-term condition(s). 

### 2.5. Analysis of Survey Results 

Several assumptions were checked before performing multiple regression analysis, namely to ensure that there was no violation of the assumptions on normality, linearity, and multicollinearity. Accordingly, it was found that the relationship between the independent variables and dependent variables was linear; there was no multicollinearity in the data; the values of residuals were independent and uncorrelated; the values of the residuals were normally distributed; there were no influential cases biasing the model. 

Multiple regression was used to analyze the relationships between the main constructs of MRQ (V3). First, the intention to reuse medicines in the future construct was treated as the dependent (outcome) variable, and the three direct measures of attitude, subjective norm, and PBC constructs were each treated as the independent (predictor) variables. We also examined the relationship between the indirect and the direct measures using bivariate correlation, where each directly measured score was treated as the dependent variable, and the sum of the corresponding weighted indirect measure was treated as the independent variable. 

To avoid interpretation problems encountered in or associated with multiple regression procedures, Structural Equation Modelling (SEM) with the standardized path coefficient was then applied using analysis of a moment structures (AMOS) SPSS to test six main hypotheses about the relationships between the main constructs of the TPB-based model. SEM allows for variables to correlate and accounts for measurement error, while multiple regression adjusts for variables in the model and assumes perfect measurement. 

SEM was also used to assess the TPB-based model’s overall goodness-of-fit. This was completed to check whether the standard relationship between the different constructs of the TPB proposed by the original model applies in relation to the data obtained in this study. These tests included chi-square, Root Mean Square Error of Approximation (RMSEA), Normalized Fit Index (NFI), Tucker Lewis Index (TLI) and the Comparative Fit Index (CFI), which are standard modification indices offered by AMOS SPSS. A new model with additional relationships between the constructs was created accordingly by calculating the modification indices (MI) using AMOS SPSS. The modification indices were checked to be at least five before the model was considered to be modified as recommended by Jöreskog and Sörbom [34]. The suggested relationships were checked carefully, and the logical relationships between constructs (i.e., only where the new relationships between the constructs made sense in relation to reusing medication as the behavior) were used to improve the model fit. 

Finally, an independent t-test (for gender) or a one-way analysis of variance (ANOVA) (for age, ethnicity, level of education, and geography) was performed to test any relationship between participant characteristics and their intention (using mean intention scores) to reuse medicines in the future, to test a further 5 hypotheses:

**Hypothesis 7** **(H7).** There will be a difference in the intention to reuse medicines in the future according to the participants’ gender.

**Hypothesis 8** **(H8).** There will be a difference in intention to reuse medicines in the future according to the participants’ age.

**Hypothesis 9** **(H9).** There will be a difference in intention to reuse medicines in the future according to the participants’ ethnicity.

**Hypothesis 10** **(H10).** There will be a difference in intention to reuse medicines in the future according to the participants’ level of education.

**Hypothesis 11** **(H11).** There will be a difference in intention to reuse medicines in the future according to the participants’ geographical location.

## 3. Results

### 3.1. Description of Findings

**Figure 2:**
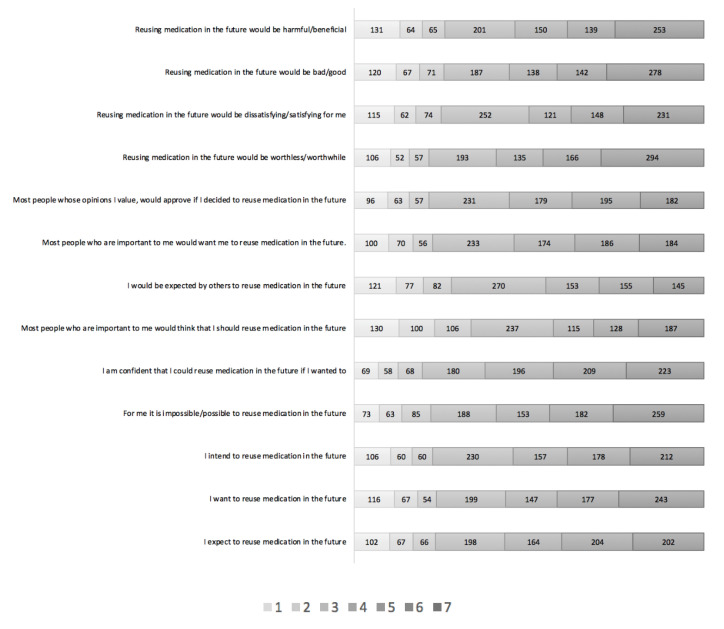
The distribution of responses to the direct questions and intention questions on the Likert scales. Key: Responses on each Likert scale, with seven indicating strongest agreement/pro-medicines-reuse sentiment and 1 indicating strongest disagreement/anti-medicines-reuse sentiment.

The majority of respondents intended to (i.e., scored 5 or more on the Likert scale, with 7 being strongly agree) (*n* = 547; 54.5%; Mean 4.67, SD 1.90), wanted to (*n* = 567; 56.5%; Mean 4.69, SD 1.98) or expected to (*n* = 570; 56.5%; Mean 4.67, SD 1.90) reuse medication in the future, while 19.7%, 19.8% and 22.9%, respectively, were unsure (scored 4 on the Likert scale) and 23.4%, 23.6% and 22.5%, respectively, disagreed (3 or less on the Likert scale, with 1 being strongly disagree) (see Figure 2). 

In terms of the direct measures of attitude, the majority of respondents (i.e., scored 5 or more on the Likert scale, with 7 being strongly agree) thought reusing medication in the future would be beneficial (*n* = 542; 54%; Mean 4.60, SD 2.03), good (*n* = 558; 55.6%; Mean 4.69, SD 2.03), satisfying for them (*n* = 500; 49.9%; Mean 4.56, SD 1.95), or worthwhile (*n* = 595; 59.3%; Mean 4.87, SD 1.97), while 20%, 18.6%, 25.1% and 19.2%, respectively, were unsure (scored 4 on the Likert scale), and 25.9%, 25.7%, 25% and 21.4%, respectively, disagreed (scored 3 or less on the Likert scale, with 1 being strongly disagree) (see Figure 2). 

In terms of the direct measures of subjective norm, the majority of respondents thought most people whose opinion they value would approve if they decided to (*n* = 542; 54%; Mean 4.64, SD 1.83), most people important to them would want them to (*n* = 553; 54.1%; Mean 4.60, SD 1.85), they would be expected by others to (*n* = 453; 45.2%; Mean 4.30, SD 1.85), or that most people important to them would think that they should (*n* = 430; 42.9%; Mean 4.23, SD 1.98) reuse medication in the future, while 23%, 23.2%, 26.9% and 23.6%, respectively, were unsure, and 21.5%, 22.5%, 27.9% and 33.5%, respectively, disagreed (see Figure 2). 

In terms of the direct measures of PBC (measuring self-efficacy), the majority of respondents felt confident they could reuse medication in the future if they wanted to, (*n* = 628; 62.6%; Mean 4.89, SD 1.78), or thought it would be possible for them to reuse medication in the future (*n* = 594; 59.2%; Mean 4.86, SD 1.86), while 17.9%, and 18.7%, respectively, were unsure, and 19.4%, and 22%, respectively, disagreed (see Figure 2). 

**Figure 3:**
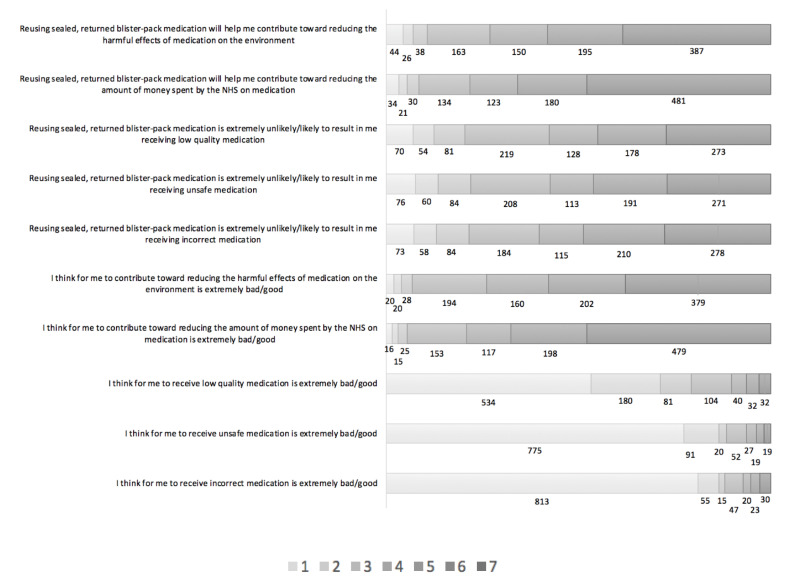
The distribution of responses to the indirect questions about behavioral beliefs and outcome evaluation, on the Likert scales. Key: Responses on each Likert scale, with 7 indicating strongest agreement/best outcome evaluation and 1 indicating strongest disagreement/worst outcome evaluation.

In terms of the indirect measures of attitude, the majority of respondents thought reusing medication will help them contribute toward reducing the harmful effects of medication on the environment (*n* = 732; 73%; Mean 5.57, SD 1.48) [with 73.9% agreeing this was good] or toward reducing the amount of money spent by the NHS on medication (n = 784; 78.2%; Mean 5.84, SD 1.43) [with 79.2% agreeing this was good], but the majority also thought that reusing medication is likely to result in them receiving low-quality medication (*n* = 579; 57.7%; Mean 5.84, SD 1.63) [with 79.3% agreeing this was bad], unsafe medication (*n* = 575; 57.3%; Mean 6.40, SD 1.36) [with 88.3% agreeing this was bad], or incorrect medication (*n* = 603; 60%; Mean 6.40, SD 1.46) [with 88% agreeing this was bad], while 16.2%, 13.4%, 21.8%, 20.7% and 18.3%, respectively, were unsure, and 10.8%, 8.5%, 20.4%, 21.9%, and 21.4%, respectively, disagreed (see Figure 3).

**Figure 4:**
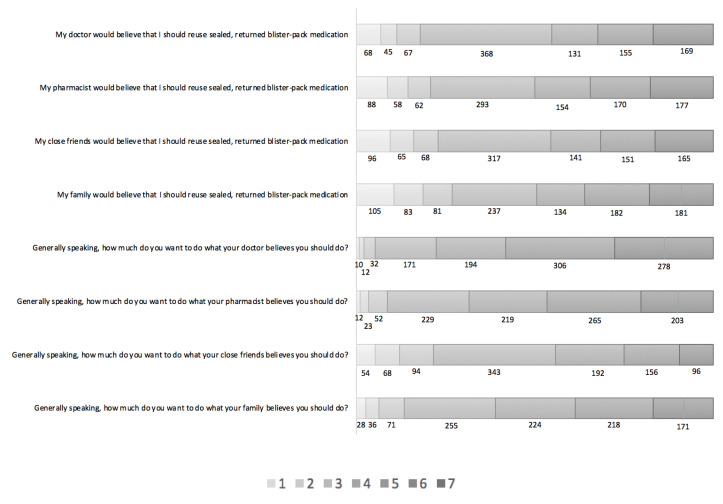
The distribution of responses to the indirect questions about normative beliefs and motivation to comply on 7-point Likert scales. Key: Responses on each Likert scale, with 7 indicating strongest agreement/motivation to comply and 1 indicating strongest disagreement/least motivation to comply.

In terms of the indirect measures of subjective norm, the majority of respondents thought that their doctor (*n* = 455; 45.4%; Mean 4.58, SD 1.67), pharmacist (*n* = 501; 50%; Mean 4.58, SD 1.77), close friends (*n* = 457; 45.6%; Mean 4.45, SD 1.79), or family (*n* = 497; 49.6%; Mean 4.48, SD 1.90) would believe they should reuse medication in the future, while 36.7%, 29.2%, 31.6% and 23.7%, respectively, were unsure, and 17.9%, 20.7%, 22.8%, and 26.8%, respectively, disagreed (see Figure 4). Overall, 77.6% agreed they would generally want to do what their doctor says, 68.5% what their pharmacist says, 44.3% what their close friends say, and 61.1% what their family says. 

**Figure 5:**
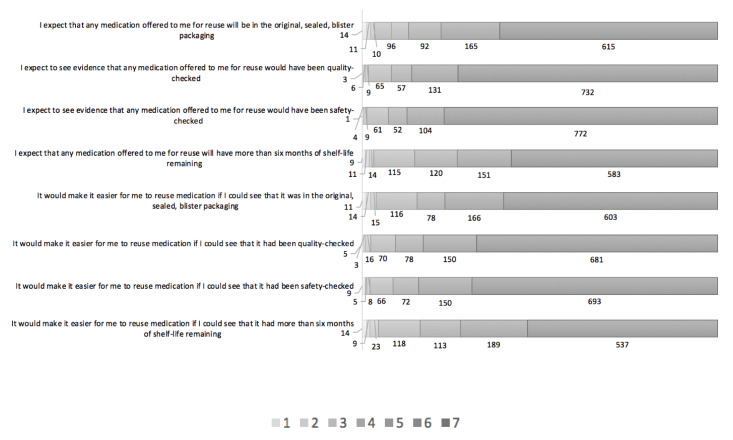
The distribution of responses to the indirect questions about control beliefs and power of control factors on 7-point Likert scales. Key: Responses on each Likert scale, with 7 indicating strongest agreement and 1 indicating strongest disagreement with the statement.

In terms of the indirect measures of PBC, the majority of respondents expected any medication offered to them for reuse will be in the original, sealed, blister packaging (*n* = 872; 86.9%; Mean 6.19, SD 1.29), would have been quality-checked (*n* = 920; 91.7%; Mean 6.48, SD 1.03), or safety-checked (n = 928; 92.5%; Mean 6.55, SD 0.97), will have more than six months of shelf-life remaining (*n* = 854; 85.1%; Mean 6.10, SD 1.29), while 9.6%, 6.5%, 6.1% and 11.5%, respectively, were unsure, and 3.5%, 1.8%, 1.4%, and 3.4%, respectively, disagreed (see Figure 5). On the whole, 84.4% agreed it would make it easier for them to reuse medication if they could see that it was in the original, sealed, blister packaging, 90.6% if it had been quality-checked, 91.2% if safety-checked, and 83.6% if it had more than six months of shelf-life remaining (see Figure 5). 

### 3.2. Regression Analysis

As shown in Table 1, intentions to reuse medicines in the future based on attitudes, subjective, norm, and PBC, returned a statistically significant regression equation: F (3, 999) = 920.645, *p* < 0.001, with an R square of 0.734 (i.e., the three independent variables accounted for 73.4% of the variance in intention to reuse medicines in the future). In addition, each of the direct measures of attitude, subjective norm, and PBC about the behavior was a positive and (statistically) significant predictor of intentions to reuse medicines in the future (B = 0.212, *p* < 0.001), (B = 0.497, *p* < 0.001), and (B = 0.326, *p* < 0.001), respectively (see Table 1). 

The correlation between the indirect and the direct measures were all statistically significant (*p* < 0.001) with a correlation between behavioral beliefs and attitude (β 0.591), and normative beliefs and subjective norm (β 0.582) being good, and the correlation between control beliefs and PBC being poor (β 0.219).

### 3.3. Construction of a TPB-based Model and Hypothesis Testing

**Figure 6:**
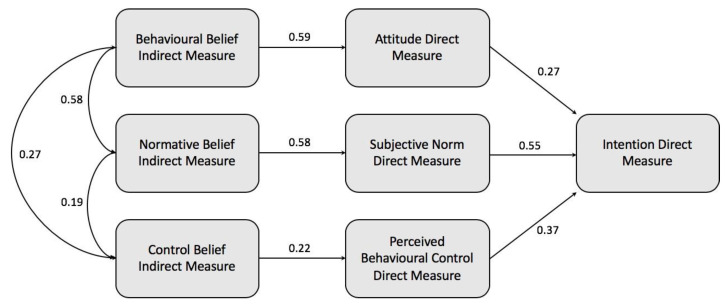
The TPB model created using SEM with standardized path analysis results.

As shown in Table 2, SEM with the standardized path coefficient returned a positive and statistically significant relationship between each of the direct measures of attitude, subjective norm, and PBC, and intention to reuse medicines in the future, upholding the first three hypotheses. Also, there were positive and statistically significant relationships between behavioral beliefs and attitude toward reusing medicines in the future, between normative beliefs and subjective norms about reusing medicines in the future, and between control beliefs and PBC about reusing medicines in the future, upholding hypotheses four to six. Figure 6 shows the complete TPB-based model using SEM with standardized path analysis. 

### 3.4. Model Modification

An additional set of tests on the model (Table 3), however, showed the assumed relationships to be a poor fit in terms of their predictive power (i.e., to predict intention to reuse medicines), necessitating the exploration of other, potentially stronger relationships between the constructs. 

The use of MI suggested 11 new relationships between the construct of the TPB model as presented in Table 4. Five of these relationships, emboldened in Table 4, were judged by the authors to be logical in relation to medicines reuse and therefore added sequentially to the model to improve the fit (Table 5). 

Briefly, the AMOS model analysis indicated that the chi-square would drop dramatically and other model values would also improve if a path was drawn from subjective norms to attitude, which seemed reasonable: people’s own attitudes could be affected by their perception of the opinion of key people in their lives, as reported in other studies using TPB [35,36]. Second, the AMOS model indicated a further improvement if a path was drawn from subjective norms to PBC; the idea that people’s confidence to reuse medication in the future could reasonably be influenced by the opinion of key people. Third, the AMOS model indicated model values would again improve if a path was drawn from behavioral beliefs to PBC; the idea that people’s self-confidence to reuse medication in the future could also be influenced by their individual beliefs about the behavior (e.g., how they might save the environment). Fourthly, the AMOS model indicated another improvement if a path is drawn from PBC to attitude; the idea that a person’s confidence to reuse medicines would influence their attitude to reuse medicines. Finally, the AMOS model indicated the model values would also improve if a path is drawn from behavioral beliefs to the subjective norm; the idea that someone’s own specific beliefs about the value of reusing medicines would influence what they considered people important to them would also believe. 

**Figure 7:**
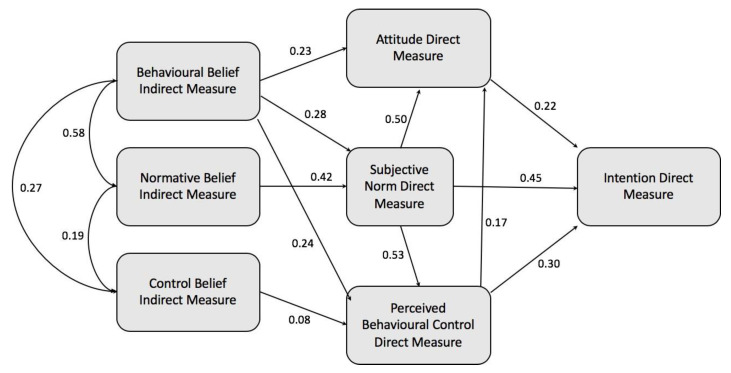
TPB model after modification showing the new relationships between the constructs.

The modified model is shown in Figure 7. Although the value of the standardized path coefficient for subjective norms reduced from 0.55 to 0.45 (*p* < 0.001, *n* = 1003) as a result of the new relationships between the construct, it remained the strongest predictor of intention to reuse medicines compared to the attitude and PBC constructs. 

### 3.5. Participant Characteristics 

There was no statistically significant difference between any of the participant characteristics and the mean intention score, leading to the rejection of hypotheses 7–11 (see Table 6). 

## 4. Discussion

The data support our premise that the direct measures of attitude toward medicines reuse, subjective norms, and PBC would positively and significantly predict intentions to reuse medication in the future and that these, in turn, would be predicted by specific behavioral, normative, and control beliefs, respectively (the latter with a proviso, explained below). The three direct measures accounted for 73.4% of the variance in relation to people’s intention to reuse medicines in the future, which was statistically significant at *p* < 0.001. The construct of subjective norms was the strongest predictor of intentions to reuse medication, with PBC also predicting intentions but attitude being a less powerful predictor. The specific indirect measures showed statistically significant correlation with the respective direct measures, but the relationship between control beliefs and PBC, although statistically significant, was poor. A modified model also provided a significant path from behavioral beliefs to subjective norms and PBC, and from the subjective norm to attitudes and PBC (but further minimized the path from control beliefs to PBC). Thus, we have shown how specific beliefs about reusing medication and what people think others would expect of them, mediated in an intricate way via attitudes, subjective, norms, and PBC, work to influence intentions to reuse medication in the future. 

The convincing effect of behavioral and normative beliefs, mainly via subjective norms and PBC, on intention is worth considering. In terms of behavioral beliefs, the findings highlight the importance of creating conditions that will first, bolster people’s beliefs about the environmental and economic benefits of medicines reuse, and second, illustrate to them that medicines reuse would not expose them to additional medication-related risks. The first point could be addressed through an educational intervention while the second could reasonably be tackled through the creative use of existing systems, and the advent of new technologies, to demonstrate safety. For example, to tackle the unwanted entry of counterfeit medicines into the supply chain, the European Union has already introduced the falsified medicines directive which specifies that manufacturers should embed specific safety features on the packaging of prescription medicines [37]. This includes a physical anti-tampering device that confirms the product had remained sealed as well as a two-dimensional barcode which when scanned authenticates the product via the unique identifier. For our purposes, this technology could be repurposed to prevent the inadvertent reissuing of low quality or unsafe medicines by eliminating counterfeit/tampered with packs. There would, however, still be a gap in the market for other technologies, for example, sensors to measure and track the interaction of the storage conditions (e.g., temperature, light, humidity) with the medicinal pack when kept outside of the formal pharmacy supply chain. Sensors that work in this way could reassure people, and reasonably regulators, about the continued quality of reused medicines, with such technology likely having the most influence on experts and health professionals who might better understand them. In terms of normative beliefs, the included injunctive norms had a significant effect on intentions to engage in medicines reuse in the future, meaning that what others would say about medicines reuse is important to people. This appears to be particularly so in terms of what doctors and pharmacists would say, and therefore a norm-based intervention could focus on encouraging doctors and pharmacists to endorse medicines reuse, which again could be achieved via sensor-based technology. 

This study did not include any items relating to descriptive social norms, simply because medicines reuse is not currently a reality—accordingly, it is suggested that any future study incorporates descriptive norms if at the time medicines reuse is in place. In addition, during the validation stage, we deleted PBC items relating to controllability (i.e., situational/external factors), which is also explained by the fact that medicines reuse is not currently in place (i.e., not externally controlled), so again we would recommend that this element is revisited in any future study. Finally, we found the relationship between control beliefs and PBC to be poor, especially in the modified model. Control beliefs ask specific questions on what facilitates or impedes the uptake of a given behavior. This finding is puzzling, especially since PBC itself was found to predict intention to reuse medication in the future, albeit by mediating the effects of behavioral beliefs and subjective norms. It is possible that the predefined context (see Section 2.2) for offering medicines for reuse (encompassing the physical characteristics and quality assurance of medicines offered for reuse) was too closely aligned to the control belief questions, creating complexity by first defining the physical characteristics and quality checks as pre-requisites to medicines reuse and then asking if they are important to control issues. Nonetheless, it is also possible that the specific control factors in MRQ (V3), although valid and reliable, did not capture the entirety of relevant ideas.

Referring back to the original elicitation study [11], as well as physical characteristics and quality assurance of medicines offered for reuse, expectations about returned medicines encompassed ideas about the logistics of medicines reuse; collecting and redistributing medicines either “on-site” within a pharmacy or “off-site”, as well as incentives for taking part in medicines reuse. The logistics of collecting and redistributing medicines for reuse logically fall outside of the control of individual patients, and while we did include a control belief question about incentives, this was deleted during the validation stage. A potential limitation of this study therefore is our inability to shed light on specific control beliefs that could be addressed through future interventions. However, read alongside our discussion about behavioral beliefs, it is reasonable to suggest that demonstrating the continued quality and safety of reissued medicines should be a pre-requisite to any future medicines reuse scheme introduced in the UK.

Despite the poor relationship between the included control beliefs and PBC, the latter was nonetheless an important mediator of intentions to reuse medicines in the future. However, since our questions focus on people’s perceived confidence in their ability (self-efficacy), it is possible that what people stated on the questionnaires might not translate into reality in the future. This is a recognized problem with using hypothetical questions [38] and further highlights the importance of recognizing the interrelationship between all of the constructs in our TPB-based model so that pro-medicines reuse behaviors could be encouraged effectively in the future through a multipronged approach. 

Most of our respondents expressed pro-medicines-reuse intentions. This concurs with the recent findings of researchers in the Netherlands, who reported that 61.2% of their respondents “were willing to use medication returned unused to the pharmacy by another patient” [39]. Based on their further analyses, those authors also conclude that guaranteeing the quality of returned medicines should facilitate people’s willingness to reuse medicines. However, by their own admission, Bekker et al. [39] did not identify in-depth information on patient barriers and facilitators to medicines reuse. In contrast, our paper provides a theoretical framework with detailed insights to guide future work. 

The online MRQ distribution to a panel of participants via a market research company (Research Now^®^) allowed us to have a representative, large and national UK sample, with ease of data gathering afforded at lower costs. Although it is possible that a face-to-face survey might have allowed further explanation of relevant points to participants, the online nature of this survey allowed a large and national sample to be reached without the risk of bias that a face-to-face survey might inadvertently have introduced. This (not being face-to-face) could be a possible limitation to this study.

A systematic review that quality appraised TPB-based questionnaire studies highlighted the main problems with these to relate to sample size estimation, omitting some of the direct and indirect measures or questions on demographics, as well as lack of detail on the questionnaire development processes [40]. The current paper illustrates in detail the process of questionnaire development, including the entirety of the validity and reliability testing following the step-by-step guidelines recommended by Ajzen [26] and Francis [32]. Thus, as a strength, our paper is the first to employ the TPB and its entire framework to measure intentions to reuse medication in the future. Another strength is that our paper is the first to systematically measure people’s views on medicines reuse via a representative sample in the UK. Our work adds to the emerging global research on medicines reuse. Finally, the discussions above show the practical implications of our findings which can be taken forward by other researchers, pharmacy regulators, government policymakers, and all others looking to decrease the impact of medication waste through medicines reuse schemes.

This study is the first to highlight public perception and willingness to take part in medicines reuse using a validated questionnaire and a large representative sample in the UK setting. In this study, most people surveyed reported positive sentiments toward medicines reuse if the safety and quality assurance of reissued medicines can be shown. By using the TPB as an underpinning theoretical framework, our paper provides detailed insights that will allow others to design specific interventions for helping the public engage with medicines reuse in the future.

## 5. Conclusions

The problem of medication waste has been recognized for decades, and there is now an emerging field of study looking at medicines reuse as a plausible solution to the ensuing economic and environmental impact of this waste. As the new decade sees the rise of a global pro-environmental movement inspired by a 16-year old, it would be a mistake to dismiss the importance of ordinary people’s opinion on the topic of medicines reuse. Although we are not suggesting that people’s views alone should be the driver for change, it is nonetheless important to understand the beliefs and willingness of people in the UK to take part in medicines reuse, especially as this is a practice that already takes place in other countries including in Greece and the US.

To understand the factors leading to medicines reuse intent, we developed, validated, and used the TPB-based MRQ. Our results show most people expressing pro-medicines-reuse intentions. Our paper shows how people could be encouraged embrace medicines reuse via practical measures that illustrate the safety and quality assurance of reissued medicines, educational interventions that bolster beliefs about the pro-environmental benefits, and norm-based interventions encouraging doctors and pharmacists to endorse the practice. 

## Figures and Tables

**Table 1:** Results of multiple regression analysis of TPB constructs using both direct and indirect measures.

Predictor Variable	B	SE	Beta (β)	t	*p*
Direct Measures					
Attitude	0.212	0.025	0.217	8.545	<0.001
Subjective norm	0.497	0.029	0.445	16.900	<0.001
PBC	0.326	0.025	0.296	12.941	<0.001
N = 1003 participants, F = 920.645, df = 3, *p* < 0.001, R = 0.857, R2 = 0.734, Adjusted R2 = 0.734					
Indirect Measures					
Behavioral belief………Attitudes F = 512.301, df = 1, *p* < 0.001, R = 0.582, R2 = 0.339, Adjusted R2 = 0.339	0.024	0.001	0.591	2.18	<0.001
Normative beliefs………Subjective norms F = 512.301, df = 1, *p* < 0.001, R = 0.591, R2 = 0.349, Adjusted R2 = 0.349	0.027	0.001	0.582	22.634	<0.001
Control beliefs………PBC F = 50.507, df = 1, *p* < 0.001, R = 0.219, R2 = 0.048, Adjusted R2 = 0.047	0.013	0.002	0.219	7.107	<0.001

**Table 2:** The testing of study hypotheses relating to the TBP-based model using SEM.

Hypotheses	Standardized Path Coefficient
H1	0.27 (*p* < 0.001, *n* = 1003)
H2	0.55 (*p* < 0.001, *n* = 1003)
H3	0.37 (*p* < 0.001, *n* = 1003)
H4	0.59 (*p* < 0.001, *n* = 1003).
H5	0.58 (*p* < 0.001, *n* = 1003)
H6	0.22 (*p* < 0.001, *n* = 1003)

**Table 3:** Measures of model fit value which indicate poor model fit.

TEST	RECOMMENDED VALUE	MODEL VALUE	DEGREE OF MODEL FIT
Chi-square Chi-square/df	*p* ≥ 0.05 ≤5	1298.857 * 108.238	Poor fit
RMSEA	≤0.08	0.327	Poor fit
NFI	≥0.9	0.676	Poor fit
TLI	≥0.9	0.435	Poor fit
CFI	≥0.9	0.677	Poor fit

df = degree of freedom; * *p* ≤ 0.001.

**Table 4:** All the new relationships between the model constructs suggested by MI.

The New Relationships between the Constructs			MI
Normative belief	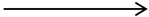	PBC	191.137
**Behavioral belief**	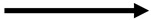	**PBC**	**241.787**
**Subjective norm**	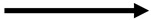	**PBC**	**430.755**
Attitude	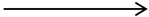	PBC	372.591
**Behavioral belief**	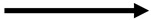	**Subjective norm**	**53.964**
PBC	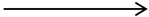	Subjective norm	238.809
Attitude	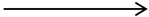	Subjective norm	312.129
Normative belief	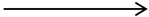	Attitude	37.007
**PBC**	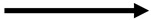	**Attitude**	**156.050**
**Subjective norm**	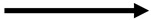	**Attitude**	**288.170**
Normative belief	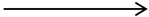	Intention	7.701

**Table 5:** Measures of model fit achieved after MIs were applied to make improvements.

TEST	RECOMMENDED VALUE	MODEL VALUE	DEGREE OF MODEL FIT
Chi-square Chi-square/df	*p* ≥ 0.05 ≤5	* 16.755 108.238	Good fit (considering a large sample)
RMSEA	≤0.08	0.037	Good fit
NFI	≥0.9	0.996	Good fit
TLI	≥0.9	0.993	Good fit
CFI	≥0.9	0.998	Good fit

df = degree of freedom; * *p* ≤ 0.001.

**Table 6:** Participant characteristics and intention to reuse medicines in the future.

HYPOTHESES	STATISTIC
H7	t = −1.506, df = 1001, *p* = 0.132
H8	F = 0.971, df = 13, 1002, *p* = 0.478
H9	F = 0.954, df = 17, 1002, *p* = 0.509
H10	F = 1.665, df = 16, 1002, *p* = 0.480
H11	F = 0.989, df = 12, 1002, *p* = 0.457

## Supplementary Materials

The following are available online at https://www.mdpi.com/2226-4787/8/4/213/s1, Figure S1: A schematic illustrating the eight steps taken in the development of the MRQ, Figure S3: The number of the items developed for each construct of the TPB, Table S4: Calculating the composite score of attitude (via the indirect measures) by multiplying scores on a unipolar scale of behavioural beliefs items by scores on a bipolar scale of outcomes evaluation (MRQ V1), Table S5: Calculating the composite score of subjective norm (via the indirect measures) by multiplying scores on a bipolar scale of normative beliefs items by scores on a unipolar scale of motivation to comply items (MRQ V1), Table S6: Calculating the composite score of PBC (via the indirect measures) by multiplying scores on a unipolar scale of control beliefs items by scores on a bipolar scale of power of control factors items (MRQ V1), Table S7: Calculating the mean of the item scores to give an overall attitude score (MRQ V1), Table S8: Calculating the mean of the item scores to give an overall subjective norm score (MRQ V1), Table S9: Calculating the mean of the item scores to give an overall PBC score (MRQ V1), Table S10: Calculating the mean of the item scores to give an overall intention score (MRQ V1), Table S11: Panel responses obtained for the content validity exercise completing before finalising MRQ V1, Table S12: The testing and modification of the MRQ (V1-3), showing the factor loadings using confirmatory factor analysis (CFA) and the test re-test reliability of the indirect measures of the MRQ items using Pearson’s correlation (r), Table S13: The internal consistency (Cronbach’s alpha) of the direct measures in MRQ V1, Table S14: The internal consistency (Cronbach’s alpha) of the direct measures in MRQ V2, Table S15: The summary of the background factors.
