# Supplementary material for: Intentions to “Reuse” Medication in the Future Modelled and Measured Using the Theory of Planned Behavior
Source: Pharmacy (Basel). 2020 Nov 12;8(4):213. doi: 10.3390/pharmacy8040213 (PMC7712992; doi:10.3390/pharmacy8040213)
Supplement: Supplementary file 1 [file pharmacy-08-00213-s001.pdf]

Intentions to ‘reuse’ medication in the future modelled and measured using the theory of planned behaviour

Supplementary materials

Figure S1 A schematic illustrating the eight steps taken in the development of the MRQ

Development of the Medicines Reuse Questionnaire (MRQ)	1) Definition of 'medicines reuse' behaviour according to *Target*, *Action*, *Context*, *Time (with reference to a previous elicitation study)*
	2) Mapping of the qualitative themes against TPB constructs *(with reference to the elicitation study)*
	3) Development of questionnaire items to measure TPB constructs *(with reference to the elicitation study)*
	4) Determination of draft questionnaire's content validity index *(working with 11 service users)*
	**Development of the first version of MRQ (V1) (*based on above steps)***
	5) Validity testing of MRQ V1 using confirmatory factor analysis and reliability testing of MRQ V1 using Cronbach's alpha *(by recruiting 46 participants to complete the MRQ V1)*
	6) Test-retest reliability of MRQ V1 using Pearson's correlation *(by asking 24 of the recruited participants to re-complete the MRQ V1 after two weeks)*
	7) Rewording of the MRQ based on the findings *(and working with 10 service users)*
	**Refinement of the MRQ to produce the second version (V2) *(based on above steps)***
	8) Validity testing of MRQ V2 using confirmatory factor analysis and reliability testing of MRQ V2 using Cronbach's alpha *(by recruiting another 46 participants to complete the MRQ V2)*
	**Refinement of the MRQ to produce the third version (V3) *(based on above steps)***

Box S2: The behaviour of interest defined for the research participants

The aim of this survey is to learn about your beliefs in relation to the concept of reusing medication. We want to know if you would personally consider reusing medication in the future. We define reusing medication as the idea that you would accept for your own personal use a prescription medication that has been previously given out to another patient but then returned to a pharmacy, where the pharmacist has verified that the medication: has been kept by the other patient for less than three months, has more than six months of shelf-life remaining, has not been tampered with, has been kept under normal storage conditions, and has been kept in an original sealed blister pack (i.e. medication strip). When we refer to reusing medication, we are interested in prescribed medication that an individual/patient may use for a long-term illness. The individual/patient would be well enough to make their own healthcare decisions.

Intentions to ‘reuse’ medication in the future modelled and measured using the theory of planned behaviour

Figure S3 The number of the items developed for each construct of the TPB

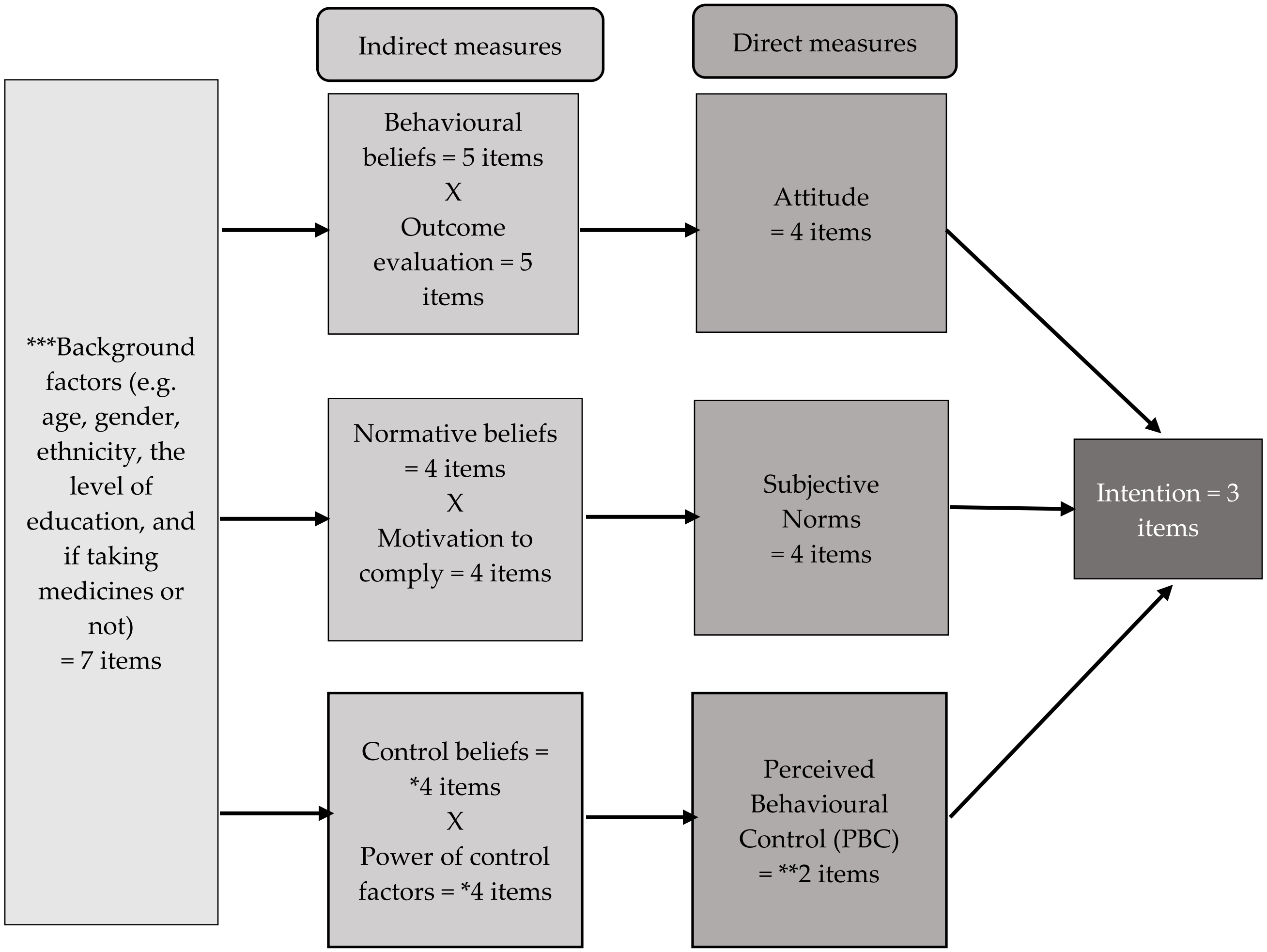

* There were 5 control belief items and 5 power of control belief items initially, but one question pair was deleted after validation so that 4 question pairs remained.

** There were 4 PBC items initially but two questions were deleted after validation so that only 2 PBC items remained

*** Religion was removed from the final version of the questionnaire

Table S4 Calculating the composite score of *attitude* (via the indirect measures) by multiplying scores on a unipolar scale of behavioural beliefs items by scores on a bipolar scale of outcomes evaluation (MRQ V1)

**Behavioural beliefs items**	**Outcomes evaluation items**
a. Reusing sealed, returned blister-pack medication will help me contribute toward reducing the harmful effects of medication on the environment.<br>*definitely disagree 1 2 3 4 5 6* ***7*** *definitely agree*	f. I think for me to contribute toward reducing the harmful effects of medication on the environment is:<br>*extremely bad -3 -2 -1 0 1 2* ***3*** *extremely good*
b. Reusing sealed, returned blister-pack medication will help me contribute toward reducing NHS drug expenditure.<br>*definitely disagree 1 2 3 4 5* ***6*** *7 definitely agree*	g. I think for me to contribute toward reducing the NHS drug expenditure is:<br>*extremely bad -3 -2 -1 0 1* ***2*** *3 extremely good*
c. Reusing sealed, returned blister-pack medication will result in me receiving low quality medication.<br>*definitely disagree 1* ***2*** *3 4 5 6 7 definitely agree*	h. I think for me to receive low quality medication is:<br>*extremely bad -3* ***-2*** *-1 0 1 2 3 extremely good*
d. Reusing sealed, returned blister-pack medication will result in me receiving unsafe medication.<br>*definitely disagree* ***1*** *2 3 4 5 6 7 definitely agree*	i. I think for me to receive unsafe medication is:<br>*extremely bad* ***-3*** *-2 -1 0 1 2 3 extremely good*
e. Reusing sealed, returned blister-pack medication will result in me receiving incorrect medication.<br>*definitely disagree* ***1*** *2 3 4 5 6 7 definitely agree*	j. I think for me to receive incorrect medication is:<br>*extremely bad* ***-3*** *-2 -1 0 1 2 3 extremely good*
For each behavioural belief, the belief score on the *extremely bad - extremely good* scale is multiplied by the relevant outcome evaluation score on the *definitely disagree - definitely agree* scale.<br>Total attitude (indirect measurement) score = (a x f) + (b x g) + (c x h) + (d x i) + (e x j).<br>For example, if the participants responded by choosing the bold numbers as above then,<br>Total attitude (A) = (7 x 3) + (6 x 2) + (2 x -2) + (1 x -3) + (1 x -3)<br>Total (A) = **+23**. Because there are 5 items, the possible range of total scores is (7 x ±3) x 5 = **-105 to +105**<br>Total A score = **+23** reflects a weak positive attitude toward reusing medication.	

Intentions to 'reuse' medication in the future modelled and measured using the theory of planned behaviour

Table S5 Calculating the composite score of *subjective norm* (via the indirect measures) by multiplying scores on a bipolar scale of normative beliefs items by scores on a unipolar scale of motivation to comply items (MRQ V1)

**Normative belief items**	**Motivation to comply items**
a. Environmentalists would believe that I should reuse sealed, returned blister-pack medication.<br>*definitely disagree* -3 -2 -1 0 1 2 **3** *definitely agree*	e. Generally speaking, how much do you want to do what environmentalists believe you should do?<br>*not at all* 1 2 3 4 5 6 **7** *very much*
b. The pharmaceutical industry would believe that I should reuse sealed, returned blister-pack medication.<br>*definitely disagree* -3 -2 **-1** 0 1 2 3 *definitely agree*	f. Generally speaking, how much do you want to do what the pharmaceutical industry believes you should do?<br>*not at all* **1** 2 3 4 5 6 7 *very much*
c. My close friends would believe that I should reuse sealed, returned blister-pack medication.<br>*definitely disagree* -3 -2 -1 0 1 **2** 3 *definitely agree*	g. Generally speaking, how much do you want to do what close friends believe you should do?<br>*not at all* 1 2 3 4 5 **6** 7 *very much*
d. My family would believe that I should reuse sealed, returned blister-pack medication.<br>*definitely disagree* -3 -2 -1 0 1 2 **3** *definitely agree*	h. Generally speaking, how much do you want to do what your family believes you should do?<br>*not at all* 1 2 3 4 5 6 **7** *very much*
For each normative belief, the belief score on the *definitely disagree - definitely agree* scale is multiplied by the relevant motivation to comply score on the *not at all - very much* scale.<br>Total subjective norm (indirect measurement) score = (a x e) + (b x f) + (c x g) + (d x h).<br>For example, if the participants responded by choosing the bold numbers as above then,<br>Total subjective norm (SN) = (7 x 3) + (-1 x 1) + (2 x 6) + (3 x 7)<br>Total (SN) = **+53**, Because there are 4 items, the possible range of total scores is (7 x ±3) x 4 = **-84 to +84**<br>Total A score = **+53** reflects moderate positive subjective norm toward reusing medicine.	

Table S6 Calculating the composite score of *PBC* (via the indirect measures) by multiplying scores on a unipolar scale of control beliefs items by scores on a bipolar scale of power of control factors items (MRQ V1)

Control beliefs items	Power of control factors items
a. I expect that any medication offered to me for reuse will be in the original, sealed, blister-packaging.<br>*definitely no* 1 2 3 4 5 **6** 7 *definitely yes*	f. It would make it easier for me to reuse medication if I could see that it was in the original, sealed, blister-packaging.<br>*strongly disagree* -3 -2 -1 0 1 **2** 3 *strongly agree*
b. I expect to see evidence that any medication offered to me for reuse would have been quality-checked.<br>*definitely no* 1 2 3 4 5 6 **7** *definitely yes*	g. It would make it easier for me to reuse medication if I could see that it had been quality-checked.<br>*strongly disagree* -3 -2 -1 0 1 2 **3** *strongly agree*
c. I expect to see evidence that any medication offered to me for reuse would have been safety-checked.<br>*definitely no* 1 2 3 4 5 6 **7** *definitely yes*	h. It would make it easier for me to reuse medication if I could see that it had been safety-checked.<br>*strongly disagree* -3 -2 -1 0 1 2 **3** *strongly agree*
d. I expect that any medication offered to me for reuse will have more than six months of shelf-life remaining.<br>*definitely no* 1 2 3 4 **5** 6 7 *definitely yes*	i. It would make it easier for me to reuse medication if I could see that it had more than six months of shelf-life remaining.<br>*strongly disagree* -3 -2 -1 0 **1** 2 3 *strongly agree*
*e. I expect to be offered some form of reward for reusing medication.<br>*definitely no* 1 2 3 **4** 5 6 7 *definitely yes*	*j. It would make it easier for me to reuse medication if I were offered some form of reward.<br>*strongly disagree* -3 -2 -1 **0** 1 2 3 *strongly agree*
For each control belief, the belief score on the *definitely no - definitely yes* scale is multiplied by the relevant power of control factor score on the *strongly disagree - strongly agree* scale.<br>Total Perceived behavioural Control (indirect measurement) score = (a x f) + (b x g) + (c x h) + (d x i) + (e x j).<br>For example, if the participants responded by choosing the bold numbers as above then,<br>Total Perceived behavioural Control (PBC) = (6 x 2) + (7 x 3) + (7 x 3) + (5 x 1) + (4 x 0)<br>Total (PBC) = **+59**, Because there are 5 items, the possible range of total scores is (7 x ±3) x 5 = **-105 to +105**<br>Total PBC score = **+59** reflects moderate positive PBC toward reusing medicine.<br>**Note*. This question pair was deleted following validation.	

Table S7 Calculating the mean of the item scores to give an overall attitude score (MRQ V1)

**Attitude**
a. Reusing medication in the future is:<br>*Harmful 1 2 3* ***4*** *5 6 7 beneficial*
b. Reusing medication in the future is:<br>*good 1* ***2*** *3 4 5 6 7 bad……………..negative endpoint*
c. Reusing medication in the future is:<br>*satisfying (for me)* ***1*** *2 3 4 5 6 7 dissatisfying (for me) ……………..negative endpoint*
d. Reusing medication in the future is:<br>*worthless 1 2 3 4 5 6* ***7*** *worthwhile*
Attitude items used a mix of positive (a and d) and negative endpoints (b and c).<br>Items with negative endpoints (b and c), were recoded so that the higher number reflects the positive attitude to the target behaviour. For example, an answer of 7 becomes a score of 1, five become a score of 3, and 4 remains 4.<br>The mean of the item scores is calculated to give an overall attitude score.<br>Mean = (4 + 6 (after recoding) + 7 (after recoding) + 7) / 4 = **6**

Intentions to 'reuse' medication in the future modelled and measured using the theory of planned behaviour

Table S8 Calculating the mean of the item scores to give an overall subjective norm score (MRQ V1)

**Subjective norm**
a. I would feel under social pressure to reuse medication in the future:<br>*strongly disagree 1 2 3* ***4*** *5 6 7 strongly agree*
b. Most people who are important to me would want me to reuse medication in the future:<br>*strongly disagree 1 2 3 4 5* ***6*** *7 strongly agree*
c. It would be expected of me to reuse medication in the future:<br>*strongly disagree 1 2 3 4 5 6* ***7*** *strongly agree*
d. Most people who are important to me would think that ------------- reuse medication in the future:<br>*I should* ***1*** *2 3 4 5 6 7 I should not……………..negative endpoint*
Subjective norm items used a mix of positive (a, b, and c) and negative endpoint (d).<br>Items with negative endpoints (d), were recoded so that the higher number reflect the positive subjective norm to reuse medicines in the future. For example, an answer of 7 becomes a score of 1, 5 becomes a score of 3, and 4 remains 4.<br>The mean of the item scores is calculated to give an overall subjective norm score.<br>Mean = (4 + 6 + 7 + 7 (after recoding)) / 4 = **6**

Intentions to 'reuse' medication in the future modelled and measured using the theory of planned behaviour

Table S9 Calculating the mean of the item scores to give an overall PBC score (MRQ V1)

**Perceived behavioural control**	
**Self-efficacy**	**Controllability**
a. I am confident that I could reuse medication in the future if I wanted to: *strongly disagree 1 2 3* ***4*** *5 6 7 strongly agree*	**c. The decision to reuse medication in the future is beyond my control: *strongly disagree 1 2 3 4 5 6* ***7*** *strongly agree*
b. For me to reuse medication in the future is: *possible 1* ***2*** *3 4 5 6 7 impossible ……………..negative endpoint*	**d. Whether I reuse medication or not in the future is entirely up to me: *strongly disagree 1 2 3 4 5 6* ***7*** *strongly agree*
PBC items used a mix of positive (a, c, and d) and negative endpoint (b).<br>Items with negative endpoints (b), were recoded so that the higher number reflect the positive PBC to reuse medicines in the future. For example, an answer of 7 become a score of 1, 5 become a score of 3, and 4 remains 4.<br>The mean of the item scores is calculated to give an overall PBC score.<br>Mean = (4 + 6 (after recoding) + 7 + 7) / 4 = **6**<br>***Note.* The two PBC questions relating to 'controllability' question pair were deleted following validation.	

Intentions to ‘reuse’ medication in the future modelled and measured using the theory of planned behaviour

Table S10 Calculating the mean of the item scores to give an overall intention score (MRQ V1)

**Intention**
a. I expect to reuse medication in the future:<br>*strongly disagree 1 2 3* **4** *5 6 7 strongly agree*
b. I want to reuse medication in the future:<br>*strongly disagree 1 2 3 4 5* **6** *7 strongly agree*
c. I intend to reuse medication in the future:<br>*strongly disagree 1 2 3 4 5 6* **7** *strongly agree*
Intention items used positive (strongly disagree - strongly agree) endpoints.<br>The mean of the item scores is calculated to give an overall intention score.<br>Mean = (4 + 6 + 7) / 3 = **5.7**

Table S11 Panel responses obtained for the content validity exercise completing before finalising MRQ V1

**Content validity question**	**Item-CVI based on responses**	**Wording of items beforehand**	**Rewording following CV**
Q1. I **understand** what this question says:<br><br>strongly agree (4), agree (3), disagree (2), strongly disagree (1)	I-CVI for responses for items 11 and 12 were 0.73 (i.e. 3 panel members disagreed they understood the question)<br><br>I-CVI for responses to item 37 was 0.64 (i.e. 4 panel members disagreed they understood the question)	11. Environmentalists think that _________ accept sealed, returned blister-pack medication.<br>*I should 1 2 3 4 5 6 7 I should not*	11. Environmentalists would believe that __________ reuse sealed, returned blister-pack medication.<br>*I should 1 2 3 4 5 6 7 I should not*
		12. Pharmaceutical industry think that _________ accept sealed, returned blister-pack medication.<br>*I should 1 2 3 4 5 6 7 I should not*	12. Pharmaceutical industry would believe that _________ reuse sealed, returned blister-pack medication.<br>*I should 1 2 3 4 5 6 7 I should not*
		37. What is your gender?<br>a. Male b. Female	37. What is your gender?<br>a. Male b. Female c. Other d. I prefer not to say
Q2. I would be able to **remember** the information needed in order to answer this question:<br><br>strongly agree (4), agree (3), disagree (2), strongly disagree (1)	I-CVI for responses ≥0.81 for all 50 items (i.e. 9 or more panel members agreed/strongly agreed they would be able to remember the information needed to answer each question)		

Q3. I would be able to **decide** which response to choose:<br><br>strongly agree (4), agree (3), disagree (2), strongly disagree (1)	I-CVI for responses ≥0.81 for all 50 items (i.e. 9 or more panel members agreed/strongly agreed they would be able to decide which response to choose for each question)		
Q4. This question is **relevant** to measuring people's beliefs about the concept of medicines reuse:<br><br>strongly agree (4), agree (3), disagree (2), strongly disagree (1)	I-CVI for responses ≥0.81 for all 50 items (i.e. 9 or more panel members agreed/strongly agreed each question was relevant to measuring people's beliefs about medicines reuse)		

Notes: Item-level Content Validity Index (I-CVI) was determined by calculating the proportion of panel members who gave a rating of 3 or 4 (agree/strongly agree) to each of the four CV questions per item. An I-CVI of ≥0.81 (9 ratings of 3 or 4 from 11 ratings) was used as the minimum acceptable value for responses to each CV question based on the work of Polit and Beck (2006). Three items not receiving the minimum acceptable value were revised.

Table S12 The testing and modification of the MRQ (V1-3), showing the factor loadings using confirmatory factor analysis (CFA) and the test re-test reliability of the indirect measures of the MRQ items using Pearson's correlation (r)

TPB construct				
Indirect measures			*Direct measures*	
**Behavioural beliefs items**	**Outcomes evaluation items**	**Composite behavioural belief**	**Attitude**	**Intention**
**Q21**. Reusing sealed, returned blister-pack medication will help me contribute toward reducing the harmful effects of medication on the environment. *definitely disagree 1 2 3 4 5 6 7 definitely agree* **V1 Test-retest reliability (r): 0.523** ***Became Q19. in MRQ (V3)***	**Q16**. I think for me to contribute toward reducing the harmful effects of medication on the environment is: *extremely bad -3 -2 -1 0 1 2 3 extremely good* **V1 Test-retest reliability (r): 0.622** ***Became Q14. in MRQ (V3)***	Q21 x Q16 **V1 Factor loading (CFA): 0.830** **V2 Factor loading (CFA): 0.525**	**Q1**. Reusing medication in the future is: *harmful 1 2 3 4 5 6 7 beneficial* **V1 Factor loading (CFA): 0.960** **V2 Factor loading (CFA): 0.934**	**Q9**. I expect to reuse medication in the future: *strongly disagree 1 2 3 4 5 6 7 strongly agree* **V1 Factor loading (CFA): 0.889** **V2 Factor loading (CFA): 0.949**
**Q22**. Reusing sealed, returned blister-pack medication will help me contribute toward reducing NHS drug expenditure. *definitely disagree 1 2 3 4 5 6 7 definitely agree*	**Q17**. I think for me to contribute toward reducing the NHS drug expenditure is: *extremely bad -3 -2 -1 0 1 2 3 extremely good*	Q22 x Q17 **V1 Factor loading (CFA): 0.831**	**Q2**. Reusing medication in the future is: *good 1 2 3 4 5 6 7 bad (negative endpoint)* **V1 Factor loading (CFA): 0.966**	**Q10**. I want to reuse medication in the future: *strongly disagree 1 2 3 4 5 6 7 strongly agree* **V1 Factor loading (CFA): 0.970**

Intentions to 'reuse' medication in the future modelled and measured using the theory of planned behaviour

**V1 Test-retest reliability (r): 0.642**<br><br>***Became Q20. in MRQ (V3)***	**V1 Test-retest reliability (r): 0.579**<br><br>***Became Q15. in MRQ (V3)***	**V2 Factor loading (CFA): 0.569**	**V2 Factor loading (CFA): 0.938**	**V2 Factor loading (CFA): 0.956**
**Q23**. Reusing sealed, returned blister-pack medication will result in me receiving low quality medication.<br>*definitely disagree* 1 2 3 4 5 6 7 *definitely agree*<br><br>**V1 Test-retest reliability (r): 0.540**<br><br>***Became Q21. in MRQ (V3)***	**Q18**. I think for me to receive low quality medication is:<br>*extremely bad* -3 -2 -1 0 1 2 3 *extremely good*<br><br>**V1 Test-retest reliability (r): 0.686**<br><br>***Became Q16. in MRQ (V3)***	Q23 x Q18<br><br>**V1 Factor loading (CFA): 0.760**<br>**V2 Factor loading (CFA): 0.777**	**Q3**. Reusing medication in the future is:<br>*satisfying (for me)* 1 2 3 4 5 6 7 *dissatisfying (for me)*<br>*(negative endpoint)*<br><br>**V1 Factor loading (CFA): 0.724**<br>**V2 Factor loading (CFA): 0.845**	**Q11**. I intend to reuse medication in the future:<br>*strongly disagree* 1 2 3 4 5 6 7 *strongly agree*<br><br>**V1 Factor loading (CFA): 0.939**<br>**V2 Factor loading (CFA): 0.895**
**Q24**. Reusing sealed, returned blister-pack medication will result in me receiving unsafe medication.<br>*definitely disagree* 1 2 3 4 5 6 7 *definitely agree*<br><br>**V1 Test-retest reliability (r): 0.685**<br><br>***Became Q22. in MRQ (V3)***	**Q19**. I think for me to receive unsafe medication is:<br>*extremely bad* -3 -2 -1 0 1 2 3 *extremely good*<br><br>**V1 Test-retest reliability (r): 0.649**<br><br>***Became Q17. in MRQ (V3)***	Q24 x Q19<br><br>**V1 Factor loading (CFA): 0.889**<br>**V2 Factor loading (CFA): 0.957**	**Q4**. Reusing medication in the future is:<br>*worthless* 1 2 3 4 5 6 7 *worthwhile*<br><br>**V1 Factor loading (CFA): 0.961**<br>**V2 Factor loading (CFA): 0.956**	

**Q25**. Reusing sealed, returned blister-pack medication will result in me receiving incorrect medication.<br>*definitely disagree 1 2 3 4 5 6 7 definitely agree*<br><br>**V1 Test-retest reliability (r): 0.506**<br><br>***Became Q23. in MRQ (V3)***	**Q20**. I think for me to receive incorrect medication is:<br>*extremely bad -3 -2 -1 0 1 2 3 extremely good*<br><br>**V1 Test-retest reliability (r): 0.750**<br><br>***Became Q18. in MRQ (V3)***	Q25 x Q20<br><br>**V1 Factor loading (CFA): 0.801**<br>**V2 Factor loading (CFA): 0.913**		
**Normative belief items**	**Motivation to comply items**	**Composite normative belief**	**Subjective norm**	
**Q26**. Environmentalists would believe that I should reuse sealed, returned blister-pack medication.<br>*definitely disagree -3 -2 -1 0 1 2 3 definitely agree*<br><br>**V1 Test-retest reliability (r): 0.216**<br><br>***Replaced by the following questions in V2:***<br>**Q26**. My doctor would believe that I should reuse	**Q30**. Generally speaking, how much do you want to do what environmentalists believe you should do?<br>*not at all 1 2 3 4 5 6 7 very much*<br><br>**V1 Test-retest reliability (r): 0.388**<br><br>***Replaced by the following questions in V2:***	Q26 x Q30<br><br>**V1 Factor loading (CFA): 0.358**<br>***Q26 and Q30 reworded in V2***<br><br>Q26 x Q30<br><br>**V2 Factor loading (CFA): 0.796**	**Q5**. I would feel under social pressure to reuse medication in the future:<br>*strongly disagree 1 2 3 4 5 6 7 strongly agree*<br><br>**V1 Factor loading (CFA): 0.262**<br>***Replaced by the following questions in V2:***<br>**Q5**. Most people whose opinions I value, would approve of my decision	

sealed, returned blister-pack medication<br>*definitely disagree -3 -2 -1 0 1 2 3 definitely agree*<br><br>***Became Q24. in MRQ (V3)***	**Q30**. Generally speaking, how much do you want to do what your doctor believes you should do?<br>*not at all 1 2 3 4 5 6 7 very much*<br><br>***Became Q28. in MRQ (V3)***		to reuse medication in the future:<br>*strongly disagree 1 2 3 4 5 6 7 strongly agree*<br><br>**V2 Factor loading (CFA): 0.759**	
**Q27**. The pharmaceutical industry would believe that I should reuse sealed, returned blister-pack medication.<br>*definitely disagree -3 -2 -1 0 1 2 3 definitely agree*<br><br>**V1 Test-retest reliability (r): 0.438**<br><br>***Replaced by the following questions in MRQ (V2):***<br>Q27. My pharmacist would believe that I should reuse sealed, returned blister-pack medication<br>*definitely disagree -3 -2 -1 0 1 2 3 definitely agree*	**Q31**. Generally speaking, how much do you want to do what the pharmaceutical industry believes you should do?<br>*not at all 1 2 3 4 5 6 7 very much*<br><br>**V1 Test-retest reliability (r): 0.381**<br><br>***Replaced by the following question in MRQ (V2):***<br>Q31. Generally speaking, how much do you want to do what your pharmacist believes you should do?	Q27 x Q31<br><br>**V1 Factor loading (CFA): 0.356**<br>***Q27 and Q31 reworded in V2***<br><br>Q27 x Q31<br><br>**V2 Factor loading (CFA): 0.843**	**Q6**.Most people who are important to me would want me to reuse medication in the future:<br>*strongly disagree 1 2 3 4 5 6 7 strongly agree*<br><br>**V1 Factor loading (CFA): 0.924**<br>**V2 Factor loading (CFA): 0.914**	

Became Q25. in MRQ (V3)	*not at all 1 2 3 4 5 6 7 very much*<br><br>***Became Q29. in MRQ (V3)***			
**Q28**. My close friends would believe that I should reuse sealed, returned blister-pack medication.<br>*definitely disagree -3 -2 -1 0 1 2 3 definitely agree*<br><br>**V1 Test-retest reliability (r): 0.663**<br><br>***Became Q26. in MRQ (V3)***	**Q32**. Generally speaking, how much do you want to do what close friends believe you should do?<br>*not at all 1 2 3 4 5 6 7 very much*<br><br>**V1 Test-retest reliability (r): 0.571**<br><br>***Became Q30. in MRQ (V3)***	Q28 x Q32<br><br>**V1 Factor loading (CFA): 1.024**<br>**V2 Factor loading (CFA): 0.618**	**Q7**. It would be expected of me to reuse medication in the future:<br>*strongly disagree 1 2 3 4 5 6 7 strongly agree*<br><br>**V1 Factor loading (CFA): 0.467**<br>**V2 Factor loading (CFA): 0.769**	
**Q29**. My family would believe that I should reuse sealed, returned blister-pack medication.<br>*definitely disagree -3 -2 -1 0 1 2 3 definitely agree*<br><br>**V1 Test-retest reliability (r): 0.533**<br><br>***Became Q27. in MRQ (V3)***	**Q33**. Generally speaking, how much do you want to do what your family believes you should do?<br>*not at all 1 2 3 4 5 6 7 very much*<br><br>**V1 Test-retest reliability (r): 0.517**	Q29 x Q33<br><br>**V1 Factor loading (CFA): 0.871**<br>**V2 Factor loading (CFA): 0.623**	**Q8**. Most people who are important to me would think that ------------- reuse medication in the future:<br>*I should 1 2 3 4 5 6 7 I should not (negative endpoint)*<br><br>**V1 Factor loading (CFA): 0.779**	

	Became Q31. in MRQ (V3)		**V2 Factor loading (CFA): 0.572**	
**Control beliefs items**	**Power of control factors items**	**Composite control belief**	**Perceived behavioural control**	
**Q34**. I expect that any medication offered to me for reuse will be in the original, sealed, blister-packaging.<br>*definitely no 1 2 3 4 5 6 7 definitely yes*<br><br>**V1 Test-retest reliability (r): 0.624**<br><br>*Became Q32. in MRQ (V3)*	**Q39**. It would make it easier for me to reuse medication if I could see that it was in the original, sealed, blister-packaging.<br>*strongly disagree -3 -2 -1 0 1 2 3 strongly agree*<br><br>**V1 Test-retest reliability (r): 0.579**<br><br>*Became Q38. in MRQ (V2)*<br><br>*Became Q36. in MRQ (V3)*	Q34 x Q39<br><br>**V1 Factor loading (CFA): 0.764**<br><br>Q34 x Q38<br>**V2 Factor loading (CFA): 0.729**	**Q12**. I am confident that I could reuse medication in the future if I wanted to:<br>*strongly disagree 1 2 3 4 5 6 7 strongly agree*<br><br>**V1 Factor loading (CFA): 0.779**<br>**V2 Factor loading (CFA): 0.943**	
**Q35**. I expect to see evidence that any medication offered to me for reuse would have been quality-checked.<br>*definitely no 1 2 3 4 5 6 7 definitely ye*<br><br>**V1 Test-retest reliability (r): 0.586**	**Q40**. It would make it easier for me to reuse medication if I could see that it had been quality-checked.<br>*strongly disagree -3 -2 -1 0 1 2 3 strongly agree*<br><br>**V1 Test-retest reliability (r): 0.530**	Q35 x Q40<br><br>**V1 Factor loading (CFA): 1.007**<br><br>Q35 x Q39	**Q13**. For me to reuse medication in the future is: *possible 1 2 3 4 5 6 7 impossible (negative endpoint)*<br><br>**V1 Factor loading (CFA): 0.878**	

Became Q33. in MRQ (V3)	*Became Q39. in MRQ (V2)*<br><br>*Became Q37. in MRQ (V3)*	**V2 Factor loading (CFA): 0.957**	**V2 Factor loading (CFA): 0.714**	
**Q36**. I expect to see evidence that any medication offered to me for reuse would have been safety-checked.<br>*definitely no 1 2 3 4 5 6 7 definitely yes*<br><br>**V1 Test-retest reliability (r): 0.596**<br><br>*Became Q34. in MRQ (V3)*	**Q41**. It would make it easier for me to reuse medication if I could see that it had been safety-checked.<br>*strongly disagree -3 -2 -1 0 1 2 3 strongly agree*<br><br>**V1 Test-retest reliability (r): 0.558**<br><br>*Became Q40. in MRQ (V2)*<br><br>*Became Q38. in MRQ (V3)*	Q36 x Q41<br><br>**V1 Factor loading (CFA): 0.923**<br><br>Q36 x Q40<br>**V2 Factor loading (CFA): 0.976**	**Q14.** The decision to reuse medication in the future is beyond my control:<br>*strongly disagree 1 2 3 4 5 6 7 strongly agree*<br><br>**V1 Factor loading (CFA): 0.258**<br><br>***Replaced by the following questions in V2:***<br>Q14. The decision to reuse medication in the future is within my control:<br>*strongly disagree 1 2 3 4 5 6 7 strongly agree*<br><br>**V2 Factor loading (CFA): -0.068**<br><br>***Therefore deleted from V3***	

**Q37.** I expect that any medication offered to me for reuse will have more than six months of shelf-life remaining.<br>*definitely no 1 2 3 4 5 6 7 definitely yes*<br><br>**V1 Test-retest reliability (r): 0.539**<br><br>***Became Q35. in MRQ (V3)***	**Q42.** It would make it easier for me to reuse medication if I could see that it had more than six months of shelf-life remaining.<br>*strongly disagree -3 -2 -1 0 1 2 3 strongly agree*<br><br>**V1 Test-retest reliability (r): 0.516**<br><br>***Became Q41. in MRQ (V2)***<br><br>***Became Q39. in MRQ (V3)***	Q37 x Q42<br><br>**V1 Factor loading (CFA): 0.434**<br><br>Q37 x Q41<br>**V2 Factor loading (CFA): 0.763**	**Q15.** Whether I reuse medication or not in the future is entirely up to me:<br>*strongly disagree 1 2 3 4 5 6 7 strongly agree*<br><br>**V1 Factor loading (CFA): 0.019**<br><br>***Replaced by the following questions in V2:***<br>Q15. Whether or not I reuse medication in the future is completely up to me:<br>*strongly disagree 1 2 3 4 5 6 7 strongly agree*<br><br>**V2 Factor loading (CFA): -0.052**<br><br>***Therefore deleted from V3***	
**Q38.** I expect to be offered some form of reward for reusing medication.	**Q43.** It would make it easier for me to reuse medication if I were	Q38 x Q43		

definitely no 1 2 3 4 5 6 7 definitely yes<br><br>**Test-retest reliability (r):**<br>**0.419**<br>***Deleted from MRQ (V2)***	offered some form of reward.<br>*strongly disagree -3 -2 -1 0 1 2 3 strongly agree*<br><br>**Test-retest reliability (r):**<br>**0.480**<br>***Deleted from MRQ (V2)***	**Factor loading (CFA):**<br>**0.026**<br>***Therefore deleted from V2***		
**Demographic questions**				
**Q44.** Are you currently taking medication for a long term condition?<br>a) Yes<br>b) No<br><br>***Became Q42. in MRQ (V2)***<br>***Became Q40. in MRQ (V3)***				
**Q45.** Choose one option that best describes your age range?<br>a) 25 or under<br>b) 26-40<br>c) 41-55<br>d) 56 or older<br>e) I prefer not to say<br><br>***Became Q43. in MRQ (V2)***<br>***Became Q41. in MRQ (V3)***				
**Q46.** What is your gender?<br>a) Male				

b) Female<br>c) Other (please specify here)<br>d) I prefer not to say<br><br>***Became Q44. in MRQ (V2)***<br>***Became Q42. in MRQ (V3)***	
**Q47.** What is your religion?<br>a) No religion<br>b) Christian (including Church of England, Catholic, Protestant and all other Christian denominations)<br>c) Muslim<br>d) Jewish<br>e) Buddhist<br>f) Hindu<br>g) Sikh<br>h) Other<br><br>***Became Q45. in MRQ (V2)***<br>***Became Q43. in MRQ (V3)***	
Q48. Choose one option that best describes your ethnicity:<br>a) White (English / Welsh / Scottish / Northern Irish/British)<br>b) White (Irish)<br>c) White (Gypsy or Irish traveller)<br>d) Any other White background, please describe below<br>e) Mixed / Multiple ethnic groups (White and Black Caribbean)<br>f) Mixed / Multiple ethnic groups (White and Black African)<br>g) Mixed / Multiple ethnic groups (White and Asian)<br>h) Any other Mixed / Multiple ethnic background, please describe here<br>i) Asian / Asian British (Indian)	

j) Asian / Asian British (Pakistani)<br>k) Asian / Asian British (Bangladeshi)<br>l) Asian / Asian British (Chinese)<br>m) Any other Asian background, please describe here<br>n) Black / Black British (African)<br>o) Black / Black British (Caribbean)<br>p) Any other Black / African / Caribbean background, please describe below<br>q) Arab<br>r) Any other ethnic group, please describe here<br>s) I prefer not to say<br><br>***Became Q46. in MRQ (V2)***<br>***Became Q44. in MRQ (V3)***
Q49. Choose one option that best describes your highest level of educational achievement:<br>a) University Higher Degree (e.g. MSc, PhD)<br>b) First degree level qualification including foundation degrees, graduate membership of a<br>c) professional Institute, PGCE<br>d) Diploma in higher education<br>e) Teaching qualification (excluding PGCE)<br>f) Nursing or other medical qualification not yet mentioned<br>g) A Level<br>h) Welsh Baccalaureate<br>i) International Baccalaureate<br>j) AS Level<br>k) Higher Grade/Advanced Higher (Scotland)<br>l) Certificate of sixth year studies<br>m) GCSE/O Level<br>n) CSE<br>o) Standard/Ordinary (O) Grade / Lower (Scotland)<br>p) Other school (including school leaving exam certificate or matriculation)

q) I prefer not to say<br>r) Other<br><br>***Became Q47. in MRQ (V2)***<br>***Became Q45. in MRQ (V3)***
Q50. If you have any comments or ideas regarding the concept of medication reuse, please share them here:<br><br>***Became Q48. in MRQ (V2)***<br>***Became Q46. in MRQ (V3)***

Intentions to ‘reuse’ medication in the future modelled and measured using the theory of planned behaviour

Table S13 The internal consistency (Cronbach’s alpha) of the direct measures in MRQ V1

TPB CONSTRUCT	CRONBACH'S ALPHA (A)	CRONBACH'S ALPHA BASED ON STANDARDIZED ITEMS	NO. OF ITEMS
**Direct measures construct and intention**	0.904	0.918	15
**Attitude**	0.935	0.944	4
**Subjective norm**	0.706	0.714	4
**Perceived behavioural control (PBC)**<br>**PBC if Q15 deleted from V1**<br>**PBC if Q14 and Q15 deleted from V1.**	**0.303**<br>**0.562**<br>0.830	0.366	4
**Intention**	0.954	0.955	3

Table S14 The internal consistency (Cronbach’s alpha) of the direct measures in MRQ V2

TPB CONSTRUCT	CRONBACH'S ALPHA (A)	CRONBACH'S ALPHA BASED ON STANDARDIZED ITEMS	N OF ITEMS
**Direct measures construct and intention**	0.931	0.930	15
**Attitude**	0.954	0.956	4
**Subjective norm**	0.818	0.820	4
**Perceived behavioural control (PBC)**	**0.425**	**0.425**	4
**Intention**	0.953	0.954	3

Intentions to ‘reuse’ medication in the future modelled and measured using the theory of planned behaviour

Table S15 The summary of the background factors

VARIABLE	FREQUENCY	PERCENTAGE
**Gender**		
**Male**	494	49.3%
**Female**	509	50.7%
**Age**		
**20-24**	10	1%
**25-29**	27	2.7%
**30-34**	47	4.7
**35-39**	45	4.5%
**40-44**	57	5.7%
**45-49**	73	7.3%
**50-54**	92	9.2%
**55-59**	133	13.3%
**60-64**	163	16.3%
**65-69**	147	14.7%
**70-74**	128	12.8%
**75-79**	60	6.0%
**80 or over**	20	2.0%
**I prefer not to say**	1	0.1%
**Ethnicity**		
**White (English / Welsh / Scottish / Northern Irish/British)**	927	92.4%
**White (Irish)**	14	1.4%
**White (Gypsy or Irish Traveller)**	2	0.2%
**Any other White background**	19	1.9%

**Mixed / Multiple ethnic groups (White and Black Caribbean)**	3	0.3%
**Mixed / Multiple ethnic groups (White and Black African)**	1	0.1%
**Mixed / Multiple ethnic groups (White and Asian)**	5	0.5%
**Any other Mixed / Multiple ethnic background**	2	0.2%
**Asian / Asian British (Indian)**	10	1%
**Asian / Asian British (Pakistani)**	3	0.3%
**Asian / Asian British (Bangladeshi)**	3	0.3%
**Asian / Asian British (Chinese)**	1	0.1%
**Any other Asian background**	2	0.2%
**Black / Black British (African)**	3	0.3%
**Black / Black British (Caribbean)**	4	0.4%
**Arab**	2	0.2%
**Other**	1	0.1%
**I prefer not to say**	1	0.1%
**Educational level**		
**University Higher Degree (e.g. MSc, PhD)**	117	11.7%
**First degree level qualification including foundation degrees, graduate membership of a professional Institute, PGCE**	179	17.8%
**Diploma in higher education**	74	7.4%
**Teaching qualification (excluding PGCE)**	20	2.0%
**Nursing or other medical qualification not yet mentioned**	22	2.2%
**A Level**	129	12.9%
**Welsh Baccalaureate**	1	0.1%
**International Baccalaureate**	5	0.5%

**AS Level**	17	1.7%
**Higher Grade/Advanced Higher (Scotland)**	21	2.1%
**Certificate of sixth year studies**	7	0.7%
**GCSE/O Level**	235	23.4%
**CSE**	40	4%
**Standard/Ordinary (O) Grade / Lower (Scotland)**	37	3.7%
**Other school (including school leaving exam certificate or matriculation)**	32	3.2%
**I prefer not to say**	27	2.7%
**Other**	40	4%
**Geographical areas**		
**East of England**	83	8.3%
**East Midlands**	57	5.7%
**London**	105	10.5%
**North East and Cumbria**	68	6.8%
**Northern Ireland**	26	2.6%
**North West**	125	12.5%
**Scotland**	86	8.6%
**South East**	151	15.1%
**South West**	95	9.5 %
**Cymru Wales**	55	5.5%
**West Midlands**	68	6.8%
**Yorkshire and the Humber**	82	8.2%
**Other**	1	0.1%
**Are you currently taking any type of medicines for your long term conditions?**		

Intentions to ‘reuse’ medication in the future modelled and measured using the theory of planned behaviour

**Yes**	867	86.4%
**No, but I have done in the past**	100	10%
**No, and I have never taken any medication for my long term condition(s)**	36	3.6%
